# Molecular MR imaging of fibrosis in a mouse model of pancreatic cancer

**DOI:** 10.1038/s41598-017-08838-6

**Published:** 2017-08-14

**Authors:** Miloslav Polasek, Yan Yang, Daniel T. Schühle, Mohammad A. Yaseen, Young R. Kim, Yu Sub Sung, Alexander R. Guimaraes, Peter Caravan

**Affiliations:** 1 A. A. Martinos Center for Biomedical Imaging, Department of Radiology, Massachusetts General Hospital and Harvard Medical School, 149 Thirteenth St., Suite 2301, Charlestown, MA 02129 USA; 2 Institute of Organic Chemistry and Biochemistry of the Czech Academy of Sciences, Flemingovo nam. 2, 16610 Prague 6, Czech Republic

## Abstract

Fibrosis with excessive amounts of type I collagen is a hallmark of many solid tumours, and fibrosis is a promising target in cancer therapy, but tools for its non-invasive quantification are missing. Here we used magnetic resonance imaging with a gadolinium-based probe targeted to type I collagen (EP-3533) to image and quantify fibrosis in pancreatic ductal adenocarcinoma. An orthotopic syngeneic mouse model resulted in tumours with 2.3-fold higher collagen level compared to healthy pancreas. Animals were scanned at 4.7 T before, during and up to 60 min after i.v. injection of EP-3533, or of its non-binding isomer EP-3612. *Ex-vivo* quantification of gadolinium showed significantly higher uptake of EP-3533 compared to EP-3612 in tumours, but not in surrounding tissue (blood, muscle). Uptake of EP-3533 visualized in T1-weighted MRI correlated well with spatial distribution of collagen determined by second harmonic generation imaging. Differences in the tumour pharmacokinetic profiles of EP-3533 and EP-3612 were utilized to distinguish specific binding to tumour collagen from non-specific uptake. A model-free pharmacokinetic measurement based on area under the curve was identified as a robust imaging biomarker of fibrosis. Collagen-targeted molecular MRI with EP-3533 represents a new tool for non-invasive visualization and quantification of fibrosis in tumour tissue.

## Introduction

In mammalian tissue, the extracellular matrix (ECM) provides integrity and mechanical support for cells, as well as performs important regulatory functions. Homeostasis of ECM is important for normal tissue function, while its dysregulation is tightly associated with diseases such as fibrosis and cancer^1, 2^. Desmoplasia, i.e. fibrosis, is typical for many solid tumours including pancreatic ductal adenocarcinoma (PDAC)^3^. The desmoplastic reaction is characterized by excessive deposition and abnormal structuring of ECM components. Type I collagen, the major protein constituent of ECM, is also dysregulated in tumour tissue and undergoes extensive remodelling^4–6^. It has been recognized that abnormal collagen distribution contributes to cancer progression and resistance to therapy in several ways. Excessive collagen increases mechanical stress that promotes tumour growth and is associated with poor prognosis^7, 8^. Linearized collagen fibres act as movement tracks for cancer cells, facilitating their migration and increasing metastatic potential^9, 10^. Dense collagen networks also impede diffusion and delivery of drugs, especially those of the macromolecular and nano-sized character^11–13^. Thus, suppression of fibrosis and collagen emerged as a promising strategy in cancer therapy, particularly in pancreatic cancer^14–19^. However, the current tools to noninvasively characterize the tumour ECM are limited. There is an unmet need for techniques that would allow visualization and quantification of collagen in tumour tissue in a non-invasive and repeatable way^6^.

Fibrosis has been traditionally assessed by biopsy and histopathological analysis. Although biopsy remains the gold standard, it is invasive and provides only a static snapshot of a small tissue sample. Second harmonic generation (SHG) imaging has been increasingly used for visualization of collagen in recent years. This two-photon microscopic technique is specific to fibrous collagens (type I and III), requires no exogenous stains, and can be adapted for *in-vivo* imaging^11, 20^. However, it is somewhat invasive in that the tumour must be exposed to light, and limited in the ability to evaluate macroscopic tissue volumes. Magnetic resonance imaging (MRI) is an attractive modality to complement biopsy and SHG imaging, as it offers combination of three-dimensional tumour coverage and deep tissue penetration.

EP-3533 is a peptide-based gadolinium-containing magnetic resonance (MR) probe targeted to type I collagen that has previously been shown to detect and stage fibrosis in animal models of liver fibrosis^21–24^, pulmonary fibrosis^25^ and cardiac fibrosis^26^. In this work we sought to determine whether EP-3533 enhanced MRI could selectively detect and quantify tumour fibrosis in an orthotopic model of PDAC that closely mimics the stroma observed in human disease^27^. A particular challenge of molecular MRI of cancer is the non-specific uptake of most molecular probes into tumours. A permeable vasculature coupled with poor lymphatic drainage results in non-specific uptake of the probe^28^. Here we validated the specificity of EP-3533 for tumour fibrosis by direct comparison with a non-binding isomer. We then showed that a model-free pharmacokinetic analysis provides a robust imaging biomarker of fibrosis that can be used to visualize and quantify fibrosis in pancreatic cancer non-invasively.

## Results

### Characterization of the animal model

**Figure 1:**
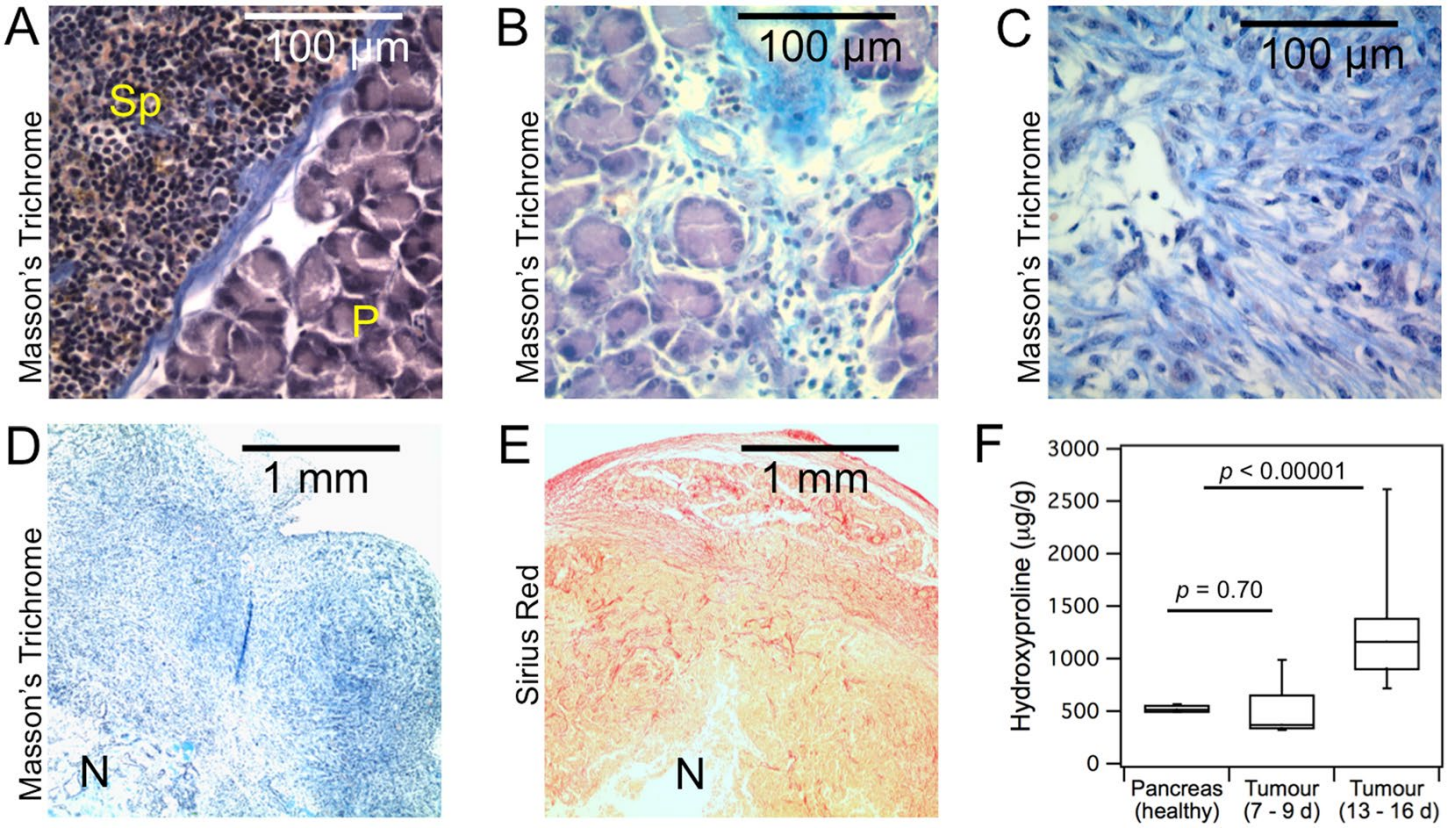
Histology and collagen quantification in pancreatic tumours and adjacent pancreas and spleen in a murine PDAC model. (**A**–**C**), high magnification images (400x) of tissue stained with Masson’s Trichrome (collagen in blue): (**A**) healthy pancreas (P) and spleen (Sp), (**B**) early tumour (day 8), (**C**) mature tumour (day 14). (**D**–**E**) low magnification (40x) images of approximately ¼ of a whole mature tumour showing heterogeneous distribution of collagen: (**D**) Masson’s Trichrome staining (collagen in blue), (**E**) Picro-Sirius red (collagen in red). N = necrosis. (**F**) quantitative analysis of hydroxyproline (surrogate for collagen) in healthy pancreas (sham control, n = 5), early tumours (days 7–9, n = 9) and mature tumours (days 13–16, n = 18).

The cancer cell line used in this animal model resulted in an aggressive tumour growth in the pancreas. To establish the best window for imaging we investigated the disease at two different time points. Early tumours (7–9 days from cell implantation) were relatively small (3–5 mm in diameter) and in some cases the boundaries could not be confidently distinguished from the surrounding pancreas (during the dissection and/or in MR images). Histological analysis showed only modest increases in positive staining for collagen in early tumours compared to healthy pancreas (Fig. 1A,B). This was in agreement with quantitative analysis of collagen (hydroxyproline) that showed no significant difference between healthy pancreas and early tumours, although a higher variation in collagen level was observed for the latter (healthy pancreas: 523 ± 14 μg/g, early tumours: 493 ± 76 μg/g, *p* = 0.70). At later times (13–16 days from cell implantation), the disease advanced into stiff, palpable tumours (~10 mm in diameter). These mature tumours manifested with prominent fibrosis as observed by strong positive staining for collagen (Fig. 1C–E). The distribution of collagen within the tumour mass was highly heterogeneous (Fig. 1D–E). Distinct necrotic areas, low in collagen compared to the rest of the tumour, were observed in most of the mature tumours. Quantitative analysis revealed that collagen content varied substantially between individual tumour specimens, and was on average 2.3-fold higher compared to healthy pancreas (1224 ± 111 μg/g vs. 523 ± 14 µg/g, *p* < 10^−5^) (Fig. 1F). Collagen was also elevated by 25% in the pancreas that was surgically separated from tumours compared to pancreas from sham control animals (682 ± 42 μg/g, *p* = 0.03).

### Identification of tumours in MR images

**Figure 2:**
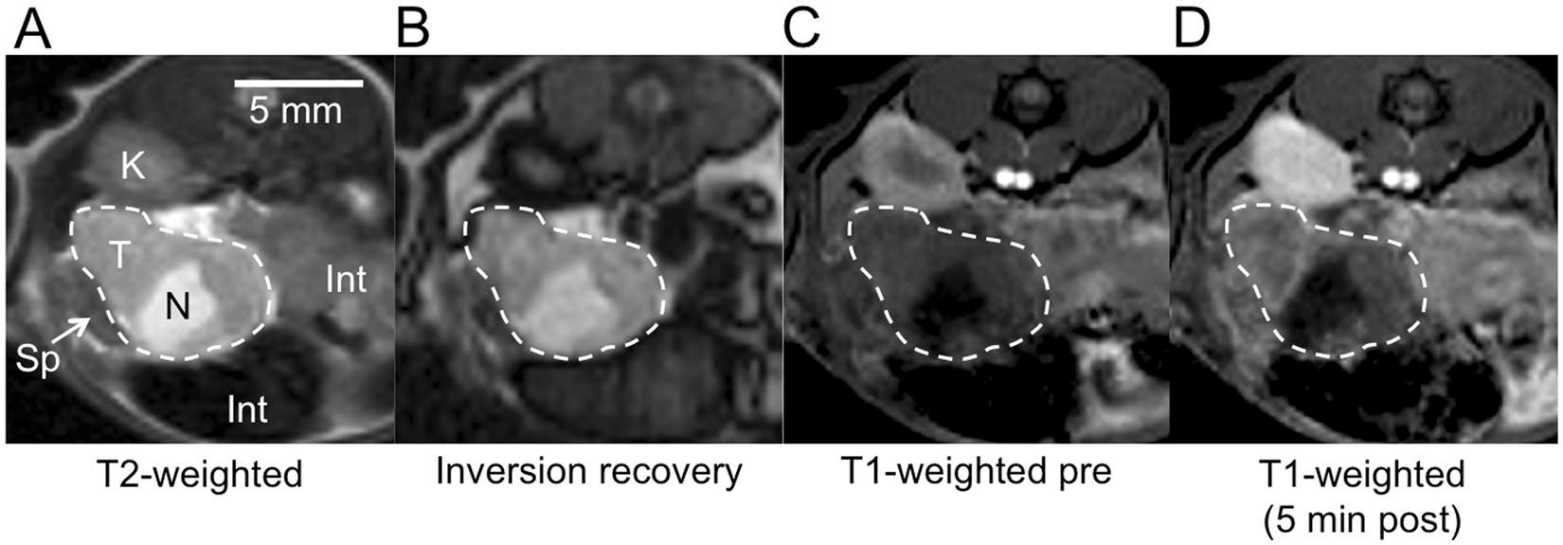
Axial MR images used to identify and characterize PDAC tumours in the mouse abdomen. Tumour is outlined. **(A**) T2-weighted image. T = tumour, N = necrosis, Sp = spleen, K = kidney, Int = intestine. (**B**) Pre-injection inversion recovery image (TR/TE/TI = 3200 ms/9.6 ms/487 ms). (**C**) Pre-injection T1-weighted image shows little contrast between tumour and surrounding tissue. (**D**) T1-weighted image at 5 min after injection of the collagen-targeted probe shows improved contrast and heterogeneous signal enhancement within the tumour.

Several imaging sequences were used to confidently define regions of interest for image analysis (Fig. 2). Good contrast between the tumour and surrounding tissue was achieved with T2-weighted and inversion recovery images (Fig. 2A,B). Distinct necrotic areas near the tumour centre were often apparent from the T2-weighted images as hyper-intense regions (Fig. 2A). In the pre-injection T1-weighted MR images the tumours appeared relatively homogeneous and, with the exception of hypo-intense necrosis, showed little contrast to the surrounding tissue (Fig. 2C). Shortly after probe injection the tumour-to-background contrast improved, with heterogeneous signal enhancement throughout the tumour mass (Fig. 2D).

### Correlation between spatial distribution of collagen in PDAC and MRI enhanced with EP-3533

**Figure 3:**
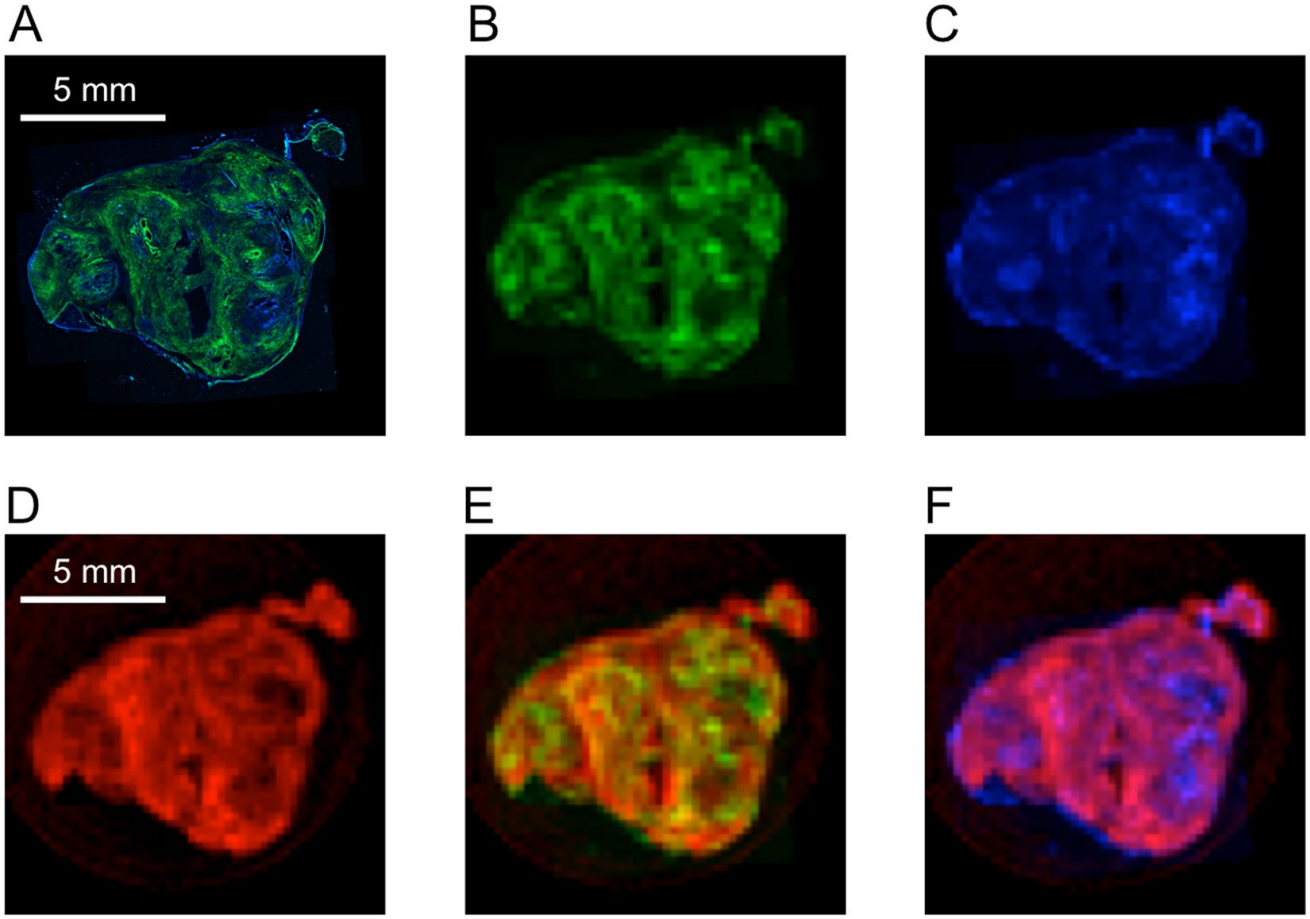
Comparison of two-photon microscopy autofluorescence and second harmonic generated (SHG) images with *ex-vivo* EP-3533 enhanced MR images of a tumour specimen. (**A**) High-resolution two-photon micrograph of a 30 μm tumour slice showing overlay of SHG image of collagen (green) and autofluorescence (blue). (**B**) SHG collagen image rendered at the same resolution as *ex-vivo* MRI. (**C**) Autofluorescence rendered at the same resolution as *ex-vivo* MRI. (**D**) T1-weighted *ex-vivo* EP-3533 enhanced MR image (animal sacrificed 80 min post-injection) of the tumour slice matched to panels (**A**–**C**). (**E**) Image overlay of EP-3533 enhanced MRI (red) with SHG image of collagen (green). (**F**) Image overlay of EP-3533 enhanced MRI (red) with autofluorescence image (blue).

To estimate how *in-vivo* EP-3533 enhanced MR images compared to the spatial distribution of collagen within the tumour volume, we performed some *ex-vivo* imaging studies. An *ex-vivo* T1-weighted MR image of a tumour that was previously imaged *in-vivo* after EP-3533 injection was compared to a collagen image obtained with SHG imaging on a 30 μm thick tumour section. Autofluorescence images that carried no information about collagen were acquired simultaneously. In agreement with histology, the SHG images revealed highly heterogeneous distribution of collagen throughout the tumour mass (Fig. 3A). Similar patterns were observed also in the *ex-vivo* EP-3533 enhanced MR images (Fig. 3D). The EP-3533 enhanced regions in MRI showed an excellent overlap with collagen-rich regions in the SHG image (Fig. 3E). However, this relationship was not reciprocal and some regions with high collagen content were not enhanced in MRI. The likely reason for this is poor perfusion and limited probe delivery. Contrary to the SHG image, the autofluorescence showed no overlap with the probe-enhanced areas in MRI (Fig. 3F).

### MRI with collagen-targeted (EP-3533) and control (EP-3612) probes

Animals with mature tumours were imaged prior to, during, and up to 60 min after *i*.*v*. injection (10 μmol/kg) of a collagen-targeted MR probe (EP-3533) or a non-binding control probe (EP-3612). Some animals were scanned consecutively with both probes in randomized order (n = 5; n = 2 received EP-3533 first, n = 3 received EP-3612 first), with a 24 h delay between imaging sessions to insure elimination of the first injected probe (for EP-3533, the residual dose found in tumours at 24 h was only 13% of the value found at 80 min post-injection; see Table S1). Altogether, imaging data sets were obtained for tumour tissue with EP-3533 (n = 8) and EP-3612 (n = 7), and for healthy pancreas (sham control animals) with EP-3533 (n = 3).

**Figure 4:**
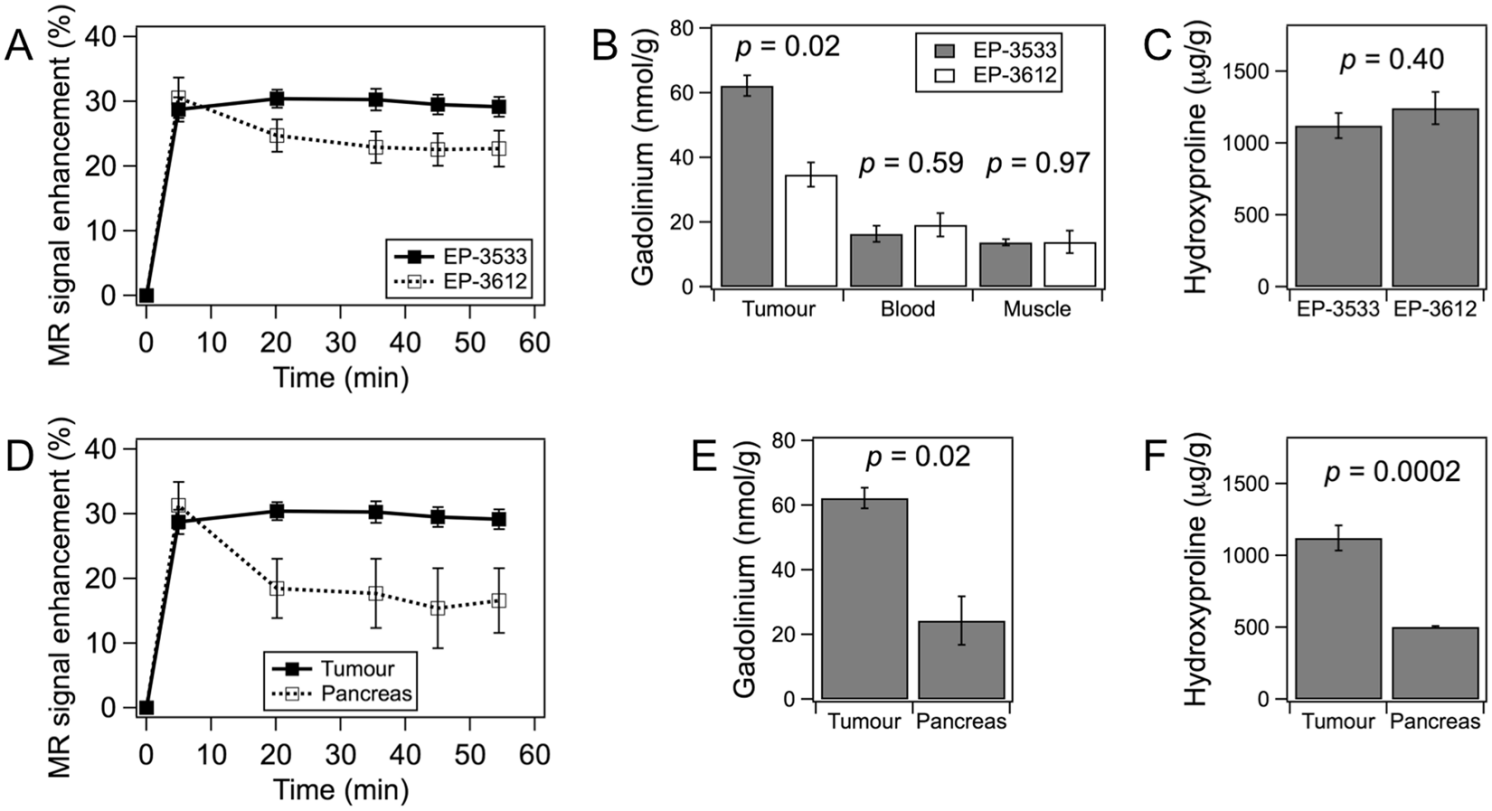
Dynamic contrast enhancement of tumours and pancreas with EP-3533 and EP-3612, Gd biodistribution and tissue hydroxyproline content in PDAC tumours and adjacent tissue. (**A**) Average MR signal enhancement relative to baseline (time = 0 min) in well-perfused tumour regions with EP-3533 (filled symbols, n = 8) and EP-3612 (open symbols, n = 7). (**B**) *Ex-vivo* quantification of gadolinium at 80 min post-injection shows significantly higher uptake of collagen-targeted (n = 3) probe in tumour tissue compared to control probe (n = 2), but no difference in the reference blood and muscle tissue. (**C**) Hydroxyproline content in tumour tissues imaged with EP-3533 (n = 8) or EP-3612 (n = 7). (**D**) Average MR signal enhancement relative to baseline (time = 0 min) in well-perfused tissue regions obtained with EP-3533 in tumour (n = 8) and healthy pancreas (n = 3). (**E**) *Ex-vivo* quantification of gadolinium in tumour (n = 3) or healthy pancreas (n = 3) 80 min after EP-3533 injection. (**F**) Hydroxyproline content in tumour (n = 8) and healthy pancreas (n = 3) of mice imaged with EP-3533.

The time courses of signal enhancement in tumour for both probes are shown in Fig. 4A. At the earliest time point after injection (5 min) both probes provided comparable signal enhancement (not significant, *p* = 0.64). This was followed by a gradual decrease of the signal enhancement with the non-binding control probe EP-3612. Differently, the signal enhancement continued increasing up to 35 min after injection of the collagen-targeted probe EP-3533, and then showed a modest decrease. Overall, EP-3533 provided higher signal enhancement in tumour compared to EP-3612 for all time points except 5 min post-injection. At 55 min post-injection, tumour signal enhancement was 29% higher with targeted probe compared to control. Quantitative *ex-vivo* analysis of gadolinium further confirmed higher uptake of EP-3533 than EP-3612 in tumour tissue (EP-3533: 62.3 ± 3.2; EP-3612: 34.7 ± 3.8 nmol/g; *p* = 0.02), while similar Gd levels were observed in blood and muscle for both probes (Fig. 4B). There was no significant difference in tumour collagen levels between the animals imaged with EP-3533 or EP-3612 (EP-3533 group: Hyp = 1121 ± 88 μg/g; EP-3612 group: Hyp = 1244 ± 112 μg/g; *p* = 0.40; Fig. 4C). The uptake of EP-3533 was further compared for tumour tissue (elevated collagen) and healthy pancreas from sham control animals (normal collagen level). Comparable signal enhancement in tumour and pancreas was observed at 5 min post-injection (*p* = 0.58) but this was followed by a rapid washout from healthy pancreas (Fig. 4D). This was in accordance with *ex-vivo* quantification of gadolinium (tumour: 62.3 ± 3.2; healthy pancreas: 24.3 ± 7.5 nmol/g; *p* = 0.02; Fig. 4E). The two tissue types also significantly differed in collagen level (Fig. 4F).

### Approximation of collagen-specific probe uptake from the area under the curve (AUC)

**Figure 5:**
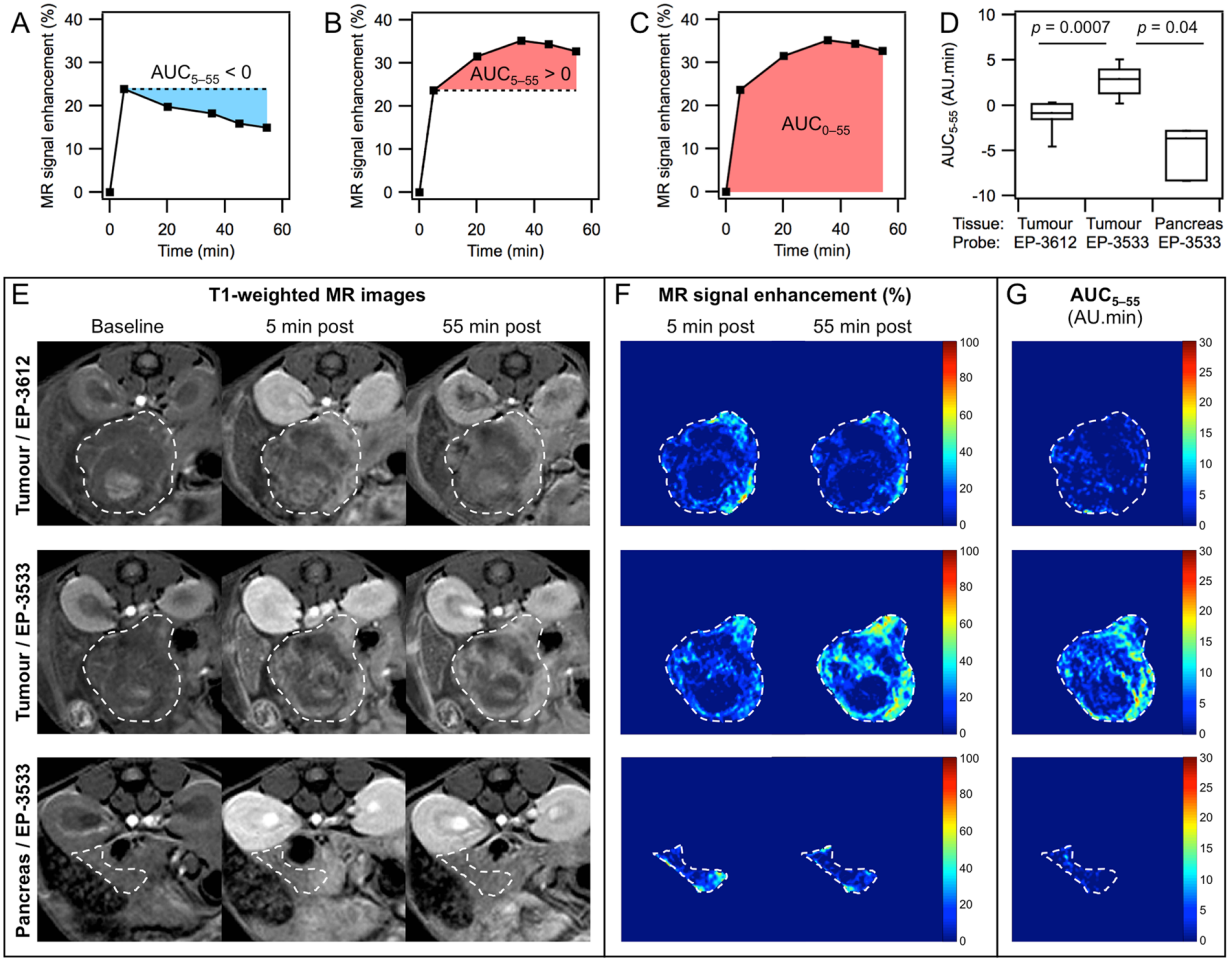
Area under the curve analyses with EP-3533 and EP-3612 enhanced MR. (**A**–**C**) Time-courses of MR signal enhancement in well-perfused regions of a single tumour showing the methods used to calculate the area under the curve (AUC_5–55_ or AUC_0–55_): (**A**) negative AUC_5–55_ (blue) obtained with EP-3612; (**B**) positive AUC_5–55_ (red) obtained with EP-3533 in the same animal as in (**A**); (**C**) AUC_0–55_ with EP-3533. (**D**) AUC_5–55_ values obtained for tumour with EP-3533 (n = 8), tumour with EP-3612 (n = 7) and healthy pancreas with EP-3533 (n = 3). (**E**) T1-weighted MR images at baseline (left), 5 min post injection (middle) and 55 min post injection (right) of a tumour imaged with EP-3612 (top row) or with EP-3533 (middle row), and for healthy pancreas imaged with EP-3533 (bottom row). The top and middle row are images of the same animal imaged 24 h apart. Bottom row is a sham control animal. Tumour and healthy pancreas are outlined. (**F**) MR signal enhancement maps for the tissue in (**E**) showing sustained enhancement at 55 min post injection with EP-3533 in tumour (middle row), but not with EP-3612 in tumour (top row) or EP-3533 in healthy pancreas (bottom row). (**G**) AUC_5–55_ maps generated from the time dependent enhancement for the tissue shown in (**E**,**F**) indicating large areas of the tumour showing positive AUC_5–55_ with EP-3533 (middle) but not with the non-binding probe EP-3612 (top) or in healthy pancreas with EP-3533 (bottom).

Figures 5A and B demonstrate typical time courses of signal enhancement in tumour observed when the control probe EP-3612 (A) and collagen-targeted EP-3533 (B) were compared in the same animal, separated in time by 24 h. We analysed this data in two ways. Since we had demonstrated that the signal enhancement at 5 min after probe injection was predominantly due to distribution and was the same for EP-3533 and EP-3612, we used this 5 min data point as a reference to calculate an area under the curve in the time window 5–55 min (AUC_5–55_) for each voxel. A mean AUC_5–55_ was then calculated for the whole tumour volume. Figure 5A and B show that the AUC_5–55_ values could be either positive or negative, depending on the profile of the curve. This is notably different from the usual meaning of the area under the curve (in this work AUC_0–55_), where the reference is taken from the baseline image and the values are thus always positive (Fig. 5C). Figure 5D compares the AUC_5–55_ obtained with 10 μmol/kg doses of probes for these categories: tumour with EP-3612, tumour with EP-3533, and healthy pancreas with EP-3533. In the tumour tissue, the average AUC_5–55_ values with EP-3533 were consistently positive (2.66 ± 0.58 AU.min), while for EP-3612 they were almost exclusively negative (−1.12 ± 0.63 AU.min). The difference between AUC_5–55_ of the two probes was statistically highly significant (*p* = 0.0007). The AUC_5–55_ values obtained with EP-3533 in healthy pancreas were exclusively negative (−4.97 ± 1.71 AU.min) and statistically significantly different from tumours (*p* = 0.04, Fig. 5D). The difference between tumours with EP-3612 and healthy pancreas with EP-3533 was not statistically significant (*p* = 0.14).

Figure 5E–G shows a series of representative T1-weighted MR images and the corresponding calculated maps of relative signal enhancement and AUC_5–55_. The top and middle rows of images depict a best-matched cross-section of the same tumour specimen imaged with EP-3612 (top) and EP-3533 (middle). The bottom row shows images of the pancreas of a healthy animal imaged with EP-3533. At 5 min post-injection in tumour, both EP-3612 and EP-3533 provided heterogeneous increase in the MR signal that was comparable in its intensity and localization within the tumour mass, as is apparent from the T1-weighted images (E) and signal enhancement maps (F). Compared to 5 min post-injection, the MR signal and signal enhancement in tumour at 55 min post-injection decreased with the non-binding EP-3612 probe, but further increased with collagen-targeted EP-3533. The difference between the two probes in tumour tissue is even more pronounced in the AUC_5–55_ maps that were windowed to show positive AUC_5–55_ values (G). With EP-3612 most of the tumour volume remains dark (negative AUC_5–55_). On the contrary, EP-3533 provides clear distinct regions that display strongly positive AUC_5–55_ values. The behaviour of EP-3533 in the pancreas of healthy mice (panels E-G, bottom row) was consistent with low specific binding of the probe, showing only modest signal enhancement at 5 min post-injection (F) that decreased at 55 min. In consequence, the AUC_5–55_ map of pancreas with EP-3533 shows mostly negative values and is dark (G). Additional T1-weighted MR images of tumours imaged with EP-3533 are provided in Figure S1.

### AUC5–55 measurement as an imaging biomarker of tumour fibrosis

**Figure 6:**
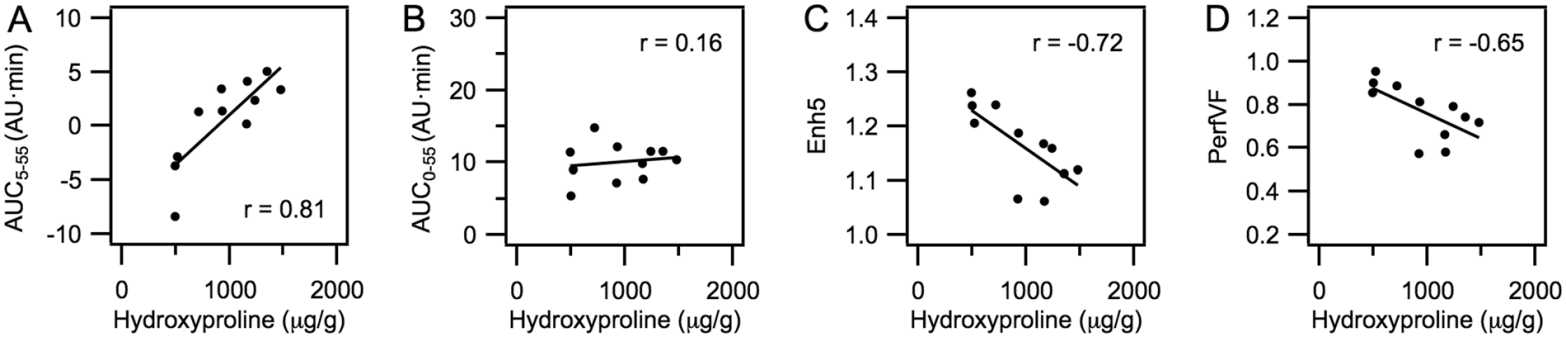
Correlations between total tissue collagen (hydroxyproline) and MRI measurements obtained with EP-3533 (n = 11). (**A**) Area under the curve with 5 min post-injection as reference (AUC_5–55_); (**B**) area under the curve with the time of injection as reference (AUC_0–55_); (**C**) MR signal enhancement at 5 min post-injection (Enh5); (**D**) volume fraction of tissue that is well perfused (PerfVF).

Figure 6A shows a good correlation (r = 0.81) between AUC_5–55_ and total tissue collagen (tumour and normal pancreas). The correlation was strong despite the fact that the tissue specimens greatly differed in perfusion characteristics and hence in the amount of probe reaching the tissue. Conversely, the full area under the curve, AUC_0–55_, displayed a poor (r = 0.16) correlation with tissue collagen (Fig. 6B). Apparently, by using the MR signal enhancement at 5 min post injection as a reference, AUC_5–55_ compensates for the differences in probe delivery due to tissue perfusion. A simple measure of signal enhancement at the last imaging time point (tumour and normal pancreas) also correlated positively with tissue hydroxyproline, but this correlation was weaker (r = 0.46) than when using AUC_5–55_. We also correlated other MR measurements with tissue hydroxyproline. Figure 6C shows that signal enhancement measured at 5 min post-injection (Enh5) negatively correlated with hydroxyproline (r = −0.72). A similar negative correlation (r = −0.65) was observed for the fraction of well-perfused tissue volume (PerfVF, Fig. 6D) compared with hydroxyproline. PerfVF was calculated as the fraction of tissue volume where the signal enhancement at 5 min post-injection surpassed a pre-defined threshold value. The Enh5 and PerfVF measurements strongly reflect perfusion of the tissue, and the negative correlation indicates that tumour perfusion worsens with increasing fibrosis.

## Discussion

PDAC is notable for the intense fibrotic reaction associated with the tumour, known as the “desmoplastic reaction”, which influences tumour survival and progression in a complex manner. Desmoplastic stroma is characterized by up to 3-fold increase in collagen (predominantly composed of Type I collagen and, to a lesser degree, type III collagen) compared with the normal pancreas^29–31^ and is also host to proliferating myofibroblasts (pancreatic stellate cells), immune, and vascular cells^32^. The stiff, fibrotic tumour microenvironment may hold the key to understanding the nature of PDAC chemoresistance through its role in regulating growth, immune response, dissemination, and limiting blood flow to the tissue^32–37^. Currently, no non-invasive tools for specifically monitoring the desmoplastic response exist, which hinders efficient translation of preclinical observations to approved therapies that are desperately needed by PDAC patients.

Here we showed in a mouse model of PDAC that the collagen-targeted MR probe EP-3533 can specifically detect and quantify Type I collagen, a protein that tightly correlates with the extent of desmoplastic reaction and with poor prognosis. Molecular imaging in cancer is confounded by the leaky vasculature and poor lymphatic drainage within tumours which leads to a non-specific enhancement with almost all imaging probes. Separating the specific, collagen-bound signal from non-specific distribution is challenging. One approach would be to employ a kinetic model to describe the time-dependent tumour signal after probe injection^38^. We took a simpler, model-free approach based on the comparison of EP-3533 with its size- and pharmacokinetic-matched negative control probe EP-3612. We observed that the initial pattern and extent of tumour enhancement was very similar immediately after injection. This is expected since the signal at these early time points will be dominated by distribution. At later time points, as unbound probe washes out of the tumour the observed signal will have a stronger contribution from the collagen-bound probe. Quantifying the AUC of the tumour signal from 5 min post injection (non-specific, distribution signal) to 55 min post injection was a useful biomarker of tumour fibrosis. The AUC_5–55_ values for the non-binding probe were almost exclusively negative indicating washout from the tumour, while the AUC_5–55_ values for EP-3533 were consistently positive. Considering tumour and healthy pancreas tissue, this simple metric correlated very well with hydroxyproline content, a biochemical measure of fibrosis.

The source of the positive AUC_5–55_ values with EP-3533 may be contributed by two factors. First, EP-3533 may accumulate in collagen-rich tissue as long as its concentration in blood is in favour of binding over detachment. Consistent with this, the blood half-life of EP-3533 in mice was previously determined as 19 min^22^. Additionally, we speculate that signal at early time points may be subject to partial volume loss because the probe is restricted in location. As the probe diffuses into the tumour, this partial volume effect is diminished and signal increases.

Other collagen-targeted probes have been described such as those based on collagen-adhesion protein^39^. A benefit of EP-3533 is its small size, which should result in faster and deeper penetration into tumour tissue compared to high molecular weight probes. There have been other indirect MR approach to assess tumour fibrosis such as through activity of matrix metalloproteinases^40^, magnetization transfer MRI^41^ or diffusion-weighted MRI^42^. Differently, EP-3533 specifically binds to type I collagen, which is directly related to fibrosis. We note that molecular MR with EP-3533 could be combined with magnetization transfer or diffusion weighted MRI, and it is possible that a combined readout could be an even more sensitive measure of tumour fibrosis.

To date, results of “anti-stromal therapy” to enhance the delivery of chemotherapeutics to PDAC by breaking down the stromal barrier have been mixed. Targeting the sonic hedgehog pathway with a smoothened inhibitor reduced type I collagen levels, decreased the desmoplasia reaction, increased intratumoural vascular density, and improved the delivery of gemcitabine, leading to prolonged survival in mice^14^, but these preclinical results did not translate to human trials^43^. However, drugs that target the desmoplastic stroma still have high potential to benefit PDAC patients. One promising mechanism to enhance therapeutic drug delivery involves use of recombinant PEGylated hyaluronidase (PEGPH20) to degrade the hyaluronic acid (HA) within the desmoplastic stroma, thus decompressing blood vessels and improving flow^37, 44, 45^. Recombinant hyaluronidase is currently undergoing clinical trials (e.g., ClinicalTrials.gov number NCT01839487) and preliminary data in patients with previously untreated stage IV pancreatic cancer suggested promising efficacy, especially in patients with a high HA content^46, 47^. Stromal collagen burden was also shown to be an important variable in eliciting increased vascular perfusion and drug delivery in animals treated with the angiotensin II receptor inhibitor losartan^17, 48^. Losartan increased perfusion through reduction in stromal collagen, reduction in hyaluronan, and decreased expression of pro-fibrotic signals such as transforming growth factor-β1 and Type I collagen expression. Importantly, these effects occurred only in collagen rich tumours, suggesting that non-invasive assessment of collagen burden could be beneficial in treatment planning during clinical trials (e.g., NCT01821729).

Noninvasive monitoring of changes in tumour stroma will be valuable in assessing treatment efficacy. For combination therapies that combine an anti-stromal therapy with a conventional chemotherapy, molecular imaging of tumour fibrosis could be used to guide the timing of the chemotherapy regimen, i.e. to begin chemotherapy after the anti-stromal therapy has shown a reduction in fibrosis.

Here we focused on developing a technique to assess and quantify tumour fibrosis with molecular MRI. However other MR readouts could also be incorporated to further characterize the tumour and its response to treatment. For instance, the similarity in initial tumour enhancement with EP-3533 and its non-binding control EP-3612 suggests that immediate dynamic imaging with EP-3533 could be used to assess vascular permeability, while the later AUC measurement could be used to assess fibrosis. Such techniques may be used to monitor and guide therapeutic approaches, where suppression of fibrosis is part of the treatment strategy. Further studies in this direction are underway.

## Methods

### Probes and Reagents

EP-3533 comprises a ten amino acid cyclic peptide conjugated to three gadolinium moieties with affinity for type I collagen (Kd = 1.8 µM) and strong MR signal enhancement (relaxivity = 16.2 mM^−1^s^−1^ (5.4 per Gd ion) at 4.7 T). EP-3612 has an identical structure to EP-3533 except that one of the cysteine moieties is changed from L-Cys in EP-3533 to D-Cys in EP-3612. This change in chirality results in >100-fold loss in collagen affinity for EP-3612, however its relaxivity remains equivalent to EP-3533. EP-3533 and EP-3612 were synthesized according to a published procedure^49^.

### Animal model

All experiments were performed in accordance with the NIH Guide for the Care and Use of Laboratory Animals and were approved by the Massachusetts General Hospital Subcommittee on Research Animal Care. A mouse pancreatic cancer cell line, from Ptf1-Cre; LSL-Kras^G12D^; p53^Lox/+^ mice (generated from publicly available strains, Jackson Laboratory: 019378, 008179, 008462) on an inbred FVB/n strain background was used to generate tumours^27^. Cells (10^6^) in 25 uL of PBS were mixed with 25 uL of Matrigel (BD Biosciences) were directly implanted in the pancreas of wild type FVB mice and the tumours were allowed to grow to up to 10 mm in diameter in 7–16 days. Sham control animals were injected with medium containing no cells.

### MR imaging

Animals were anesthetized with isoflurane (1–2%) and continuously maintained under this anaesthesia up to the end of experiment (return to cage or sacrifice). Animals were placed in a specially designed cradle with body temperature maintained at 37 °C. The tail vein was cannulated for intravenous (*i*.*v*.) delivery of the imaging probe while the animal was positioned in the scanner. Imaging was performed at 4.7 T using a small-bore animal scanner with a custom-built volume coil. EP-3533 or EP-3612 were administered at a dose of 10 μmol/kg as 5 mM solutions in PBS. Thus 2 µL per gram of body weight was administered using a calibrated 100 µL Hamilton syringe, and accounting for the dead volume of the tubing.

A series of baseline images were acquired, a bolus the imaging probe was administered *i*.*v*. and the imaging was repeated for a period of 60 min post-injection. Animals were euthanized ca. 80 min post-injection and tissues samples were harvested for further analysis. A subset of animals underwent imaging with a second probe, for those animals a delay of 24 h was used between the imaging sessions to allow elimination of the first injected probe. In animals that received both probes, the probes were administered in randomized order. Sham control animals were imaged only with EP-3533.

Baseline MR images included T1-weighted gradient echo sequences with axial (TR/TE/flip angle = 92.6 ms/2.77 ms/35°, matrix = 144 × 144, FOV = 28.8 × 28.8 mm, ten 1-mm thick slices) and coronal orientation (TR/TE/flip angle = 105.4 ms/3.156 ms/35°, matrix = 192 × 192, FOV = 38.4 × 38.4 mm, ten 1-mm thick slices), T2-weighted rapid acquisition with refocused echo (RARE) sequence (TR/TE = 5000/40, matrix = 128 × 128, FOV 28.8 × 28.8 mm, ten 1-mm thick slices) and T1-mapping sequence (RARE inversion recovery (IR), TR/TE = 3200 ms/9.6 ms, matrix = 96 × 96, 9 inversion times from 47 to 3000 ms, RARE factor 8, single 1 mm slice). Following the injection, the axial T1-weighted sequence and T1-map were repeated at approximate times 5, 20, 35, 45 and 55 min post-injection.

For *ex-vivo* MR imaging the animal was first imaged with EP-3533 as described above. After sacrificing at 80 min post-injection, the tumour was excised and fixed with a suture in a plastic tube filled with OCT compound. *Ex-vivo* images were acquired with a T1-weighted 3D gradient echo sequence (TR/TE/flip angle = 10 ms/2.58 ms/20°, matrix = 64 × 64 × 128, FOV = 16 × 16 × 32 mm, 250 μm isotropic resolution). The tumour was then frozen and sectioned for two-photon microscopy so as to preserve the orientation.

### Image analysis

Axial T1-weighted MR images were used for the analysis. Images in DICOM format were opened in OsiriX software, regions of interests (ROIs) were drawn to outline tumours and dorsal muscle in all slices that contained the tumour. ROI masks were exported from OsiriX using CreateROIMask plugin. The DICOMs and masks were further analysed in freeware Octave. See Supplementary data for the data processing scripts. The analysis was performed on the entire tumour volume and included the following steps: First, the image intensity was normalized to average dorsal muscle signal (each time-point individually). Then, maps of signal enhancement for each post-injection time-point were calculated as: signal enhancement, SE = (signal post-injection)/(pre-injection baseline signal). Areas under the curve (AUC) were calculated from the enhancement time courses for each voxel individually before being averaged for the entire tissue volume. AUC values are expressed as SE (with arbitrary units, AU) × time (minutes) with units AU•min. No co-registration of images was applied.

Tissue perfusion was assessed based on signal enhancement at 5 min post-injection. A distinction between well- and poorly-perfused voxels was based on the median signal enhancement at 5 min of all analysed voxels (tumour + pancreas voxels of all animals). Data for both EP-3533 and EP-3612 probes were considered together. Taking the median as a threshold, a voxel was assigned as well perfused if the signal enhancement at 5 min surpassed this threshold. The well-perfused tissue volume fraction (PerfVF) was calculated for each tissue specimen as: (number of well-perfused voxels in ROI)/(total number of voxels in ROI).

### *Ex-vivo* tissue analysis

Formalin-fixed samples were embedded in paraffin, cut into 5 µm-thick sections and stained with Masson’s trichrome, Sirius red or hematoxylin and eosin (H&E) stain according to standard procedures. Quantitative analysis of hydroxyproline was used as a measure of total amount of collagen in tissue. Hydroxyproline was quantified by HPLC analysis using a reported method^50^. Gadolinium was quantified in nitric acid digests by inductively coupled plasma-mass spectrometry (ICP-MS) using dysprosium as an internal standard. Hydroxyproline and gadolinium are expressed as μg or nmol per g of wet tissue weight, respectively.

### Two-photon microscopy

Collagen content within *ex-vivo* tumour tissue slices was visualized by second harmonic generation (SHG) imaging using a commercial multiphoton imaging system (Ultima, Bruker, Middleton, WI). Unstained 30 µm-thick slices on glass slides were imaged in an epifluorescence configuration using a 4x microscope objective (Olympus XLFluor 4, 0.28 NA, Center Valley, PA). Excitation light (λ = 880 nm) was delivered using a tuneable, pulsed Titanium:Sapphire laser (100 fs pulse duration, 80 MHz repetition rate, Mai-Tai Tsunami, Spectra-Physics, Santa Clara, CA) and scanned across the field of view using galvanometer scanning mirrors. Using dichroic beamsplitters and optical bandpass filters, emitted SHG light from excited collagen and autofluorescence signal were collected through the objective, spectrally separated, and directed to separate photomultiplier tube detectors (SHG: 460 ± 25 nm, autofluorescence: 525 ± 25 nm).

For a single image, the field of view 2.35 × 2.35 mm was captured with 9.18 µm per pixel resolution. The whole tissue slice was imaged in sections by moving the sample in the xy-plane with an automated translation stage. The entire thickness of the tissue slice was imaged by collecting a depth-resolved z-stack of images at each location (9 images, 10 µm interslice distance). A complete image was obtained as a maximum projection image first for each z-stack, followed by stitching of multiple fields of view using ImageJ software with a stitching plugin^51^. Coregistration with MRI was performed in Octave by rotation and resizing of micrographs using bicubic interpolation and re-slicing of 3D MR data so as to achieve the best visual agreement between the tumour boundaries.

### Statistical analysis

All data are shown as mean ± standard error of the mean (SEM). Error bars represent SEM. Box plots show the 0 and 100 percentile (whiskers), 25 and 75 percentile (box) and median value (horizontal bar). Statistical significance was tested by unpaired two-tailed Student’s t-test (Excel), with *p* < 0.05 as significant.

### Data availability

The datasets generated during and/or analysed during the current study are available from the corresponding author on reasonable request.

## Electronic supplementary material

Supporting Information
