# Supplementary material for: Molecular MR imaging of fibrosis in a mouse model of pancreatic cancer
Source: Sci Rep. 2017 Aug 14;7:8114. doi: 10.1038/s41598-017-08838-6 (PMC5556073; doi:10.1038/s41598-017-08838-6)
Supplement: Supplementary file 1 — Supporting Information [file 41598_2017_8838_MOESM1_ESM.pdf]

Supplementary Information for

Molecular MR imaging of fibrosis in a mouse model of pancreatic cancer

Miloslav Polasek[1,2], Yan Yang[1], Daniel T. Schühle[1], Mohammad A. Yaseen[1], Young R. Kim[1], Yu Sub Sung[1], Alexander R. Guimaraes[1], Peter Caravan[1,*]

[1] A. A. Martinos Center for Biomedical Imaging, Department of Radiology, Massachusetts General Hospital and Harvard Medical School, 149 Thirteenth St., Suite 2301, Charlestown MA 02129, USA.
[2] Institute of Organic Chemistry and Biochemistry of the Czech Academy of Sciences, Flemingovo nam. 2, 16610 Prague 6, Czech Republic.

Contents:

Page 2	**Table S1.** Quantification of gadolinium in tumours and reference tissue at 80 min and 24 h post-injection of EP-3533 and EP-3612.
Page 3	**Figure S1.** T1-weighted MR images of tumours imaged with collagen-targeted probe EP-3533 and corresponding time courses of MR signal enhancement.
Page 4	General information about data processing.
Page 5 – 6	Script “read_info”
Page 7 – 9	Script “load_data”
Page 10 – 11	Script “read_mask”
Page 12	Script “normalize”
Page 13 – 16	Script “AUC_roi”
Page 17	Script “ENH_calc”
Page 18 – 19	Script “track_file”
Page 20 – 21	Script “results”

Table S1. Quantification of gadolinium in tumours and reference tissue at 80 min and 24 h post-injection of EP-3533 and EP-3612.

Probe	Time post-injection	Gd in tissue (nanomol per gram of wet tissue) Average ± SEM		
		Tumour	Blood	Muscle
EP-3533	80 min	62.3 ± 3.2	16.3 ± 2.5	13.7 ± 0.9
EP-3533	24 h	7.8 ± 2.1	0.6 ± 0.3	1.0 [a]
EP-3612	80 min	34.7 ± 3.8	19.1 ± 3.7	13.9 ± 3.5
EP-3612	24 h	8.4 ± 0.6	0.4 ± 0.1	2.2 ± 0.5

[a] Only one measurement was performed.

Figure S1. T1-weighted MR images of tumours imaged with collagen-targeted probe EP-3533 and corresponding time courses of MR signal enhancement. MR images at various time points are shown for three selected animals. The left column shows images of the same animal as in Figure 5 in the main text. The tumours are outlined. Corresponding time courses of MR signal enhancement in well-perfused tumour regions are shown at the bottom.

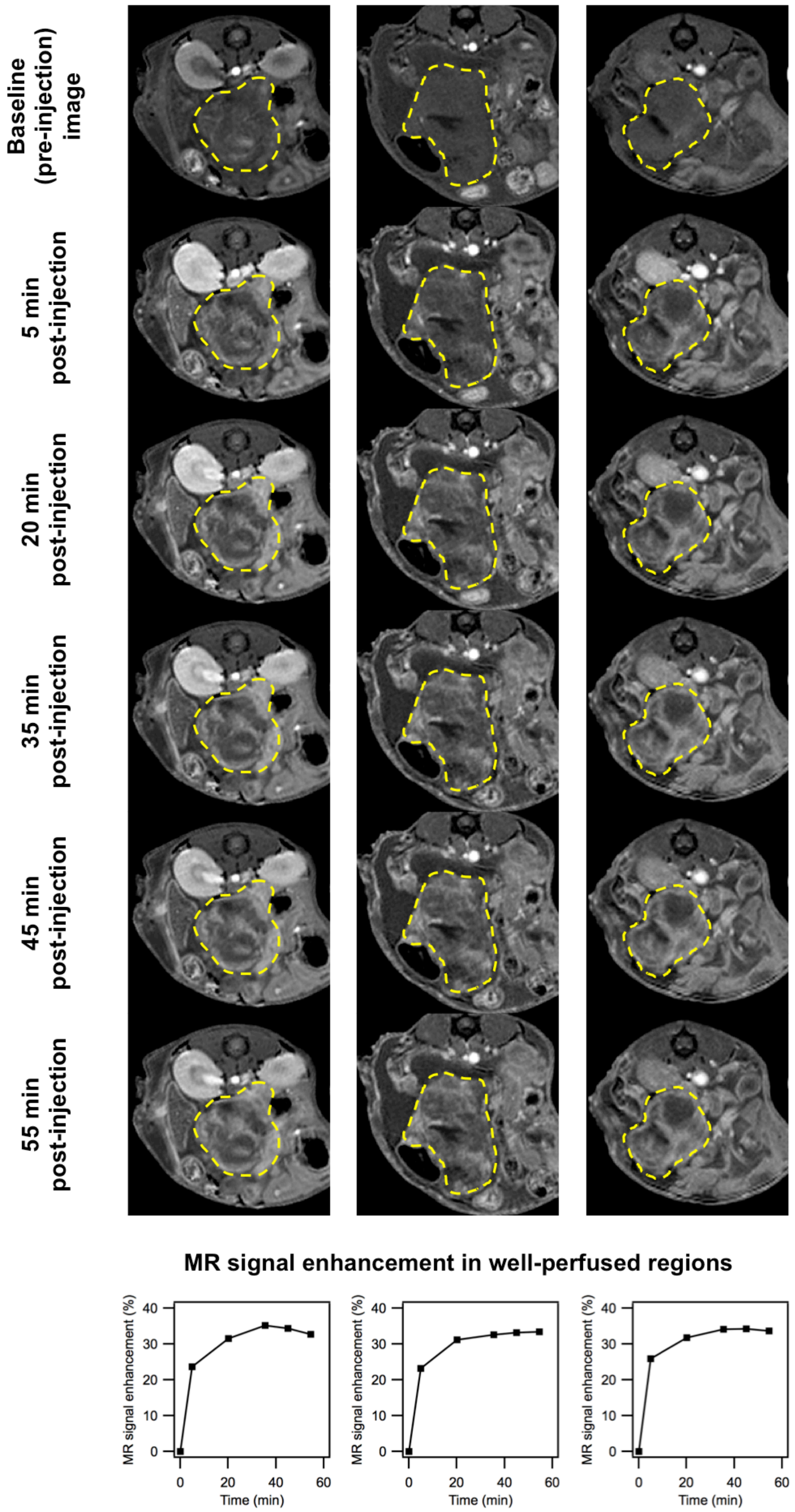

General information about data processing.

The MRI data analyzed in this paper have been exported from Bruker 4.7 T scanner in DICOM format. For processing the data, freeware Octave (https://www.gnu.org/software/octave/) was used. To read data in DICOM format with Octave it is necessary to install Dicom package created by Andy Buckle (http://wiki.octave.org/Dicom_package).

The scripts described in the following pages were written and used with Octave 3.8.1 with the following versions of packages installed:

```
Package Name   | Version |
      control *|   2.6.5 |
        dicom *|   0.1.1 |
      general *|   1.3.4 |
        image *|   2.2.2 |
miscellaneous  |   1.2.0 |
        optim  |   1.3.0 |
     parallel  |   2.2.0 |
       signal *|   1.3.0 |
       struct  |  1.0.10 |
```

Since Octave and Matlab share the same programming language, it should be possible to use the scripts also with Matlab (perhaps after minor debugging).

By running each script, some variables are created that are used by the next script. Therefore, in order to function properly, the scripts must be run in the following order:

1. read_info
2. load_data
3. read_mask
4. normalize
5. AUC_roi
6. ENH_calc
7. track_file
8. results

Script “read_info”

```
%==================start of read_info
%Written by Miloslav Polasek
%PURPOSE: To retreive information from DICOM files, such as file name
and time of data acquisition.
%RESULT: Cell array "NeatList" that stores this information and is
referred to by other scripts.


%Here define the path to data directory.
session_dir = ["put here the path to the data directory"];

%Creates a list of subdirectories.
%Subdirectories are individual experiments within the imaging session.
%Inside subdirectories are individual images, each slice separately.
biglist=dir(fullfile(session_dir,'*'));

CellAr = cell(size(biglist,1),4);
NeatList = cell(size(biglist,1),5);

%The following loop will go through all experiments within the imaging
session.
for i=1:size(biglist,1)
  scan_dir=strcat(session_dir,biglist(i).name);
  cd(scan_dir);
  clear small_list

  %Creates a list of images contained in an experiment.
  small_list=dir('*m*');

  %Stores information about the number of images (slices) in the
experiment.
  CellAr{i,1}=size(small_list,1);

  clear D_info;
  D_info=dicominfo(small_list(1).name);

  %Retreives acquisition time of the experiment.
  CellAr{i,2}=(D_info(1).Private_0008_0032);

  %Retreives name of the acquisition sequence.
  CellAr{i,3}=D_info(1).Private_0018_1030;

  %Stores the name of the subdirectory (experiment) that contains the
images.
  CellAr{i,4} = biglist(i).name;

  %Stores consecutive number of the experiment.
  NeatList{i,1} = i;
end

% Creates NeatList by sorting experiments according to acquisition time
in ascending order.
NeatList(1:size(biglist,1),2:5) = sortrows(CellAr,2);

% Cleaning temporary variables.
clear CellAr
```

```
clear small_list
clear i
clear D_info
clear scan_dir
clear biglist
cd(session_dir);
cd ..;
%===================end of read_info
```

Script “load_data”

```
%==================start of load_data
% Written by Miloslav Polasek
% PURPOSE: This script will read MRI data from DICOM files and create
% a 4-dimensional matrix, where in first 2 dimensions is an actual
image,
% these are stacked along 3rd dimension (slices), and repetitions of
the
% experiment are stacked along 4th dimension.
% It also calculates the time that passed since imaging probe was
injected
% for each experiment repetition.

% RESULT: A 4-dimensional matrix "DATA". Image intensity in each image
element
% is expressed as the numeric type "double".
% Time since probe injection (in minutes) is stored in "theseTimes".

% Here define the experiments that you wish to load by entering
% their number as defined in "NeatList". These must be repetitions
% of the same experiment with the same dimensions.
% For example, theseScans = [3,7,10] will load 3rd, 7th and 10th
experiment
% from the imaging session.
theseScans = [3,7,10,13,16,18]; %Replace these numbers.

% Here define the experiment during which imaging probe was injected.
% Typically this is some DCE (dynamic contrast enhancement) sequence.
% Enter the number of that experiment as defined in "NeatList".
% This is important for correctly calculating time since probe
injection.
injScan = [6]; % Replace this number.

% This is a delay in seconds that marks the instant of probe injection
% during the DCE experiment mentioned above. In our case it was 90
seconds
% after the start of acquisition.
timeDelay = 90; % Replace this number.

% Prepares empty variables for reading image data.
clear BB
BB=[];
DATA = [];

% Prepares empty variables for reading time information.
tempTimes = [];
theseTimes = [];

% Prepares modified version of "theseScans" for reading time
information.
theseScansMod = theseScans;
theseScansMod(1,1) = injScan;   % enters ref. number for long DCE scan
to serve as starting point


%------------------start reading DATA
for i=1:size(theseScans,2)
```

```
  scan_dir=strcat(session_dir,NeatList{theseScans(1,i),5});
  cd(scan_dir);
  clear small_list

  %Make sure in the following line that the definition of the file name
  %in parentheses is correct. This depends on the naming scheme of the
  %DICOM images that was used. In my case, the files were named
"MRImXX",
  %where XX was a 2-digit number representing consecutive MRI slices.
  % ('*') should work if MRI images are the only files in directory.
  small_list=dir('*m*');

  clear AA
  AA=[];

  %Concatenates MRI slices along 3rd dimension (3D image).
  for j=1:size(small_list,1)
    slice=dicomread(small_list(j).name);
    AA=cat(3,AA,slice);
  end

  %Concatenates the temporary 3D image along 4th dimension
(repetitions).
  BB=cat(4,BB,AA);

end

% IMPORTANT!
% To allow division operations with "DATA" it is important to remove
values
% equal to 0. They are replaced with 1. This has negligible effect on
quantitative
% results, because the meaningful values of image intensity in DICOM
are in 1000s or
% 10,000s (for 16 bit coding as in our case).
DATA = double(BB);
DATA(DATA < 1) = 1;

% Cleaning temporary variables.
clear BB
clear i
clear scan_dir
clear small_list
clear AA
clear j
clear slice

%--------------------end reading DATA

%..................start reading time info
% Retreives information about when experiment was started and
% converts it from the DICOM format to time expressed in seconds.
for i=1:size(theseScansMod,2)
    retreive = cell2mat(NeatList(theseScansMod(1,i),3));

    %The baseline experiment (before injection of the probe)
    %is by default assigned time = 0.
    TT = 0;
```

```
    TT = TT + str2double(retreive(1,1))*36000;
    TT = TT + str2double(retreive(1,2))*3600;
    TT = TT + str2double(retreive(1,3))*600;
    TT = TT + str2double(retreive(1,4))*60;
    TT = TT + str2double(retreive(1,5))*10;
    TT = TT + str2double(retreive(1,6));
    tempTimes(i,1) = TT;
end

% Calculates time in seconds since the time of probe injection.
for i=1:size(theseScansMod,2)
    theseTimes(1,i) = tempTimes(i,1) - tempTimes(1,1) - timeDelay;
end

% Converts time in seconds to time in minutes.
theseTimes = theseTimes / 60;

% The baseline experiment is assigned time = 0.
theseTimes(1,1) = 0;

% Cleaning temporary variables.
clear i
clear retreive
clear tempTimes
clear theseScansMod
clear injScan
clear TT;
clear timeDelay
%..................end reading time info
%==================end of load_data
```

Script "read_mask"

```
%==================start of read_mask
% Written by Miloslav Polasek
% PURPOSE: This script reads masks from .img files (Analyze format).
These can be
% created for example in OsiriX software by drawing region of interest
(ROI) and
% exporting with CreateROIMask plugin.
% The masks will be used in further data processing.
% The input mask data must be a matrix with the same dimensions as the
MRI image
% to be overlayed with. In general this means a 3-dimensional matrix.
% In addition, this script defines a smallest 3D box to which the
entire mask can
% fit. This is useful in order to limit certain time-consuming
calculations
% only to the data that is relevant for image analysis.
% In our case we used two masks: (1) for regions containing tumor, (2)
for regions
% containing dorsal muscle. The MRI signal in muscle will be used for
normalization
% of the MRI image intensity.
% RESULT: Two masks are created for tissue of interest (in our case
tumor) and for
% reference tissue (in our case muscle).


% Here define the path to the directory containing mask in Analyze
format (.img file).
mask_is_here = ["~/Documents/data_imaging/PancreaticCancer_masks/"
animal "_" probe "_mask/"];
cd(mask_is_here);

% Reads mask for tumor regions.
mask_file_name = 'tumor.img'; % Define the correct file name.
clear mask;
mask = analyze75read(mask_file_name);
% This will make the mask to consist of values 0 or 1.
mask(mask>0)=1;
maskT = double(mask);

% Reads mask for muscle regions.
mask_file_name = 'muscle.img'; % Define the correct file name.
clear mask;
mask = analyze75read(mask_file_name);
% This will make the mask to consist of values 0 or 1.
mask(mask>0)=1;
maskM = double(mask);


% Defines a rectangular area where the mask fits (to disregard useless
data).
% The area is defined by four indexes representing lowest and highest
position
% in columns (C) and rows (R) where mask is located. The third
dimension is
% not considered. These indexes will be used in further data analysis.
```

```
temp = sum(maskT,3);
C = sum(temp);
R = sum(temp,2);
indC1 = 0;
indC2 = size(maskT,2); % Maximal number of columns (dimension 2).
indR1 = 0;
indR2 = size(maskT,1); % Maximal number of rows (dimension 1).
foundit1 = 0;
foundit2 = 0;

% Finds indexes for columns (1st dimension).
for i=1:size(maskT,2)
  if(C(i) != 0 && foundit1 == 0)
    indC1 = i;
    foundit1 = 1;
  endif

  a = size(C,2) + 1 - i;
  if(C(a) != 0 && foundit2 == 0)
    indC2 = a;
    foundit2 = 1;
  endif
end
foundit1 = 0;
foundit2 = 0;

% Finds indexes for rows (2st dimension).
for i=1:size(maskT,1)
  if(R(i) != 0 && foundit1 == 0)
    indR1 = i;
    foundit1 = 1;
  endif

  a = size(R,1) + 1 - i;
  if(R(a) != 0 && foundit2 == 0)
    indR2 = a;
    foundit2 = 1;
  endif
end

% Cleaning temporary variables.
clear mask;
clear mask_is_here;
clear mask_file_name;
clear mask_is_here;
clear temp;
clear C;
clear R;
clear i;
clear foundit1;
clear foundit2;
clear a;
%===================end of read_mask
```

Script "normalize"

```
%==================start of normalize
% Written by Miloslav Polasek
% PURPOSE: This script will normalize the image intensity in the
original MRI data
% to the signal in reference region of interest (in our case we used
dorsal muscle).
% The normalization is done for each time point (repetition)
separately. This is
% necessary in order to correct for the drift in signal intensity over
longer
% time periods.

% RESULT: 4-dimensional matrix "DATA" where signal intensity is
normalized
% to the average signal in reference ROI.


% Extracts data according to the masks defined in "read_mask" script.
muscle = DATA;
tumor = DATA;
DATAorig = DATA; % Back up of original not-normalized image data.
% Masks and MRI image are overlaid to erase irrelevant data.
for j=1:size(DATA,4)
    muscle(:,:,:,j) = muscle(:,:,:,j) .* maskM;
    tumor(:,:,:,j) = tumor(:,:,:,j) .* maskT;
end


% Normalize image intensity for each time point (4th dimension in DATA)
separately.
for j=1:size(DATA,4)
    % Average signal intensity in reference ROI.
    signalMav(j,1) = sum(sum(sum(muscle(:,:,:,j)))) /
sum(sum(sum(maskM)));

    % Normalize image intensity.
    DATA(:,:,:,j) = DATA(:,:,:,j) / signalMav(j,1);
end


% Cleaning temporary variables.
clear i
clear j
clear signalMav
clear muscle
clear tumor
%==================end of normalize
```

Script "AUC_roi"

```
%==================start of AUC_roi
% Written by Miloslav Polasek
% PURPOSE: This script calculates the area under the curve (AUC) in two
ways.
% (1) The full AUC between time points 0 - 55 minutes. Here it is the
variable "AUC".
% In the publication it is denoted as AUC0-55.
% (2) The AUC between time points 5 - 55 minutes. Here it is the
variable "AUC2".
% In the publication it is denoted as AUC5-55.
% The values are calculated for each voxel separately. Indexes that
have been defined
% in "read_mask" script are applied here to limit the calculation only
to relevant
% regions of interest.

% RESULT: 3-dimensional maps "AUC" and "AUC2" that will be later used
to calculate
% the average area under the curve values for the entire region of
interest.


% Set this value to the last time point to be used for AUC calculation.
% In our case this was 55 minutes after probe injection.
% The actual values at this time point will be extrapolated (see
further).
timelimit = 55;

AUC = zeros(size(DATA,1),size(DATA,2),size(DATA,3)); % Full AUC (0 - 55
minutes).
AUC2 = zeros(size(DATA,1),size(DATA,2),size(DATA,3)); % AUC (5 - 55
minutes).
DATA55 = zeros(size(DATA,1),size(DATA,2),size(DATA,3)); % Extrapolated
data at time 55 min.

T = theseTimes; % For simplicity.

% These loops go through every voxel in 3D MRI data. Indexes defined in
"read_mask" script
% are applied to limit the amount of data and shorten computation time.
% First the the image intensity at 55 min is extrapolated. Then AUC
calculations are
% done using the normalized image intensities stored in "DATA", and the
extrapolated
% value at 55 min as the last data point.
% NOTES TO DATA EXTRAPOLATION:
% The exact method for extrapolation of data at 55 min depends on
availability of data
% nearest to this time point. If data exists for a time point > 55 min,
then the image
% intensity at 55 min is linearly extrapolated from the values
immediately before and
% after 55 min. If the last available time point is at < 55 min, then
the value of
% image intensity at that last point is used for 55 min time point with
no change.
```

```
% Area under the curve is calculated in parts (between each pair of
neighboring
% time points) that are then summed together.
for h=indR1:indR2 % Analysis limited by indexes to ROI.
  for j=indC1:indC2 % Analysis limited by indexes to ROI.
    for k=1:size(DATA,3) % Every slice is analyzed.
      % Linearize the repetitions for easy data access.
      D = reshape(DATA(h,j,k,:),1,numel(DATA(h,j,k,:)));

      %--------------------------------
      % Calculation of AUC (0 - 55 min)
      % The loop goes through the time points and calculates AUC
between
      % neighboring time points and stores them in variable "A".
      lastcalc = 0; % Has the end of the curve been reached? (false or
true)
      for i=1:(size(T,2))
        % Check availability of data with respect to "timelimit".
        if((i+1) <= size(T,2) && T(1,i+1) <= timelimit)
          % If true, it means that both time points are earlier than
the "timelimit"
          % and area under the curve between them can be calculated as
follows:
          A(i) = (T(1,i+1) - T(1,i)) * ((D(1,i) - D(1,1)) + (D(1,i+1) -
D(1,i))/2);

        % First case when end of curve is reached:
        elseif((i+1) <= size(T,2) && T(1,i+1) > timelimit && lastcalc
== 0)
          % If true, it means that data at time point > "timelimit". It
is therefore
          % possible to linearly extrapolate value of signal intensity
at
          % time = "timelimit" and use the value for area under the
curve calculation.
          lastcalc = 1; % The end of the curve has been reached (true).

          % Linear extrapolation of data (signal intensity) at
"timelimit".
          dataTL = D(1,i) + (D(1,i+1) - D(1,i))/(T(1,i+1) -
T(1,i))*(timelimit - T(1,i));

          % Store the extrapolated data (creating an image).
          DATA55(h,j,k) = dataTL;

          % Calculate area under the curve with extrapolated data.
          A(i) = (timelimit - T(1,i)) * ((D(1,i) - D(1,1)) + (dataTL -
D(1,i))/2);

        % Second case when end of curve is reached:
        elseif ((i+1) > size(T,2) && lastcalc == 0)
          % If true, all available data was acquired at time points <
"timelimit" and
          % linear extrapolation is not possible. Instead, the value of
signal intensity
          % at "timelimit" is set equal to the value from the last
available time point.
          lastcalc = 1; % The end of the curve has been reached (true).
```

```
          % Approximate the value at "timelimit" with the closest
preceding data.
          dataTL = D(1,i);

          % Store the approximated data (creating an image).
          DATA55(h,j,k) = dataTL;

          % Calculate area under the curve with approximated data.
          A(i) = (timelimit - T(1,i)) * ((dataTL - D(1,1)) + (dataTL -
D(1,i))/2);

        % All other calculations are skipped
        else
          A(i) = 0;
        endif
      end

      % Total area under the curve is calculated by summing the
increments.
      % The result is stored in 3D matrix.
      AUC(h,j,k) = sum(A);

      %--------------------------------
      % Calculation of AUC2 (5 - 55 min)
      % This is analogous to the calculation of AUC above, except that
the starting point
      % is the signal intensity at time 5 min. Our imaging protocol was
set up so that
      % the first MRI image after probe injection (second repetition in
DATA) was acquired
      % consistently at 5 min after probe injection. The calculation
thus starts from the
      % second repetition.
      lastcalc = 0;
      for i=2:(size(T,2))
        if((i+1) <= size(T,2) && T(1,i+1) <= timelimit)
          B(i) = (T(1,i+1) - T(1,i)) * ((D(1,i) - D(1,2)) + (D(1,i+1) -
D(1,i))/2);
        elseif((i+1) <= size(T,2) && T(1,i+1) > timelimit && lastcalc
== 0)
          lastcalc = 1;
          dataTL = D(1,i) + (D(1,i+1) - D(1,i))/(T(1,i+1) -
T(1,i))*(timelimit - T(1,i));
          B(i) = (timelimit - T(1,i)) * ((D(1,i) - D(1,2)) + (dataTL -
D(1,i))/2);
        elseif ((i+1) > size(T,2) && lastcalc == 0)
          lastcalc = 1;
          dataTL = D(1,i);
          B(i) = (timelimit - T(1,i)) * ((dataTL - D(1,2)) + (dataTL -
D(1,i))/2);
        else
          B(i) = 0;
        endif
      end
      AUC2(h,j,k) = sum(B);
      %--------------------------------
    end
```

```
  end
end


% Cleaning temporary variables.
clear h
clear i
clear j
clear k
clear A
clear B
clear D
clear lastcalc
clear dataTL
clear T
%===================end of AUC_roi
```

Script "ENH_calc"

```
%===================start of ENH_calc
% Written by Miloslav Polasek
% PURPOSE: Calculate signal enhancement relative to baseline image
% at selected time points.

% RESULT: Maps of signal enhancement at 5 minutes ("ENH") and
% at 55 minutes ("ENH2") after probe injection.


% Calculate signal enhancement at 5 minutes after probe injection
% (i.e. for second repetition)
ENH = DATA(:,:,:,2) ./ DATA(:,:,:,1);

% Calculate signal enhancement at 55 minutes after probe injection
% (i.e. for second repetition)
ENH2 = DATA55 ./ DATA(:,:,:,1);

% Cleaning temporary variables.
clear i
%===================end of ENH_calc
```

Script “track_file”

```
%==================start of track_file
% Written by Miloslav Polasek

% PURPOSE: Create a matrix "track" where each row represents an
individual
% image voxel located within the region of interest. Stores the
information
% about voxel coordinates in 3D space and the results of various
measurements
% for this voxel, namely signal enhancements at 5 and 55 minutes, and
area
% under the curve from 0 to 55 minutes and from 5 to 55 minutes. In
this way
% the data is prepared for mutual correlations, calculating averages
for ROI,
% or for applying threshold filtering.

% RESULT: Matrix "track" that can be used for further data analysis.

%Define which ROI mask will be applied.
mask = maskT;

% Initialize "track" matrix with number of rows equal to the number
% of voxels in the mask.
track = zeros(sum(sum(sum(mask))),7);

countG = 0; %Global counter

% This loop will go through all imaging data and extract values from
% positions included in the region of interest defined by the mask.
% For each valid voxel, the information is saved in one row of the
% "track" matrix, in the respective column.
for k=1:size(DATA,3) % Each slice
  count = 0; % Local counter
  for i=1:size(DATA,1) % Each row
    for j=1:size(DATA,2) % Each column

      % Checks if the position is valid and should be considered.
      if (mask(i,j,k) == 1)
        ++countG;
        track(countG,1) = i; % Row number
        track(countG,2) = j; % Column number
        track(countG,3) = k; % Slice number

        % Signal enhancement at 5 minutes after probe injection.
        track(countG,4) = ENH(i,j,k);

        % Full AUC from 0 to 55 minutes after probe injection
        track(countG,5) = AUC(i,j,k);

        % AUC from 5 to 55 minutes after probe injection
        track(countG,6) = AUC2(i,j,k);

        % Signal enhancement at 55 minutes after probe injection.
        track(countG,7) = ENH2(i,j,k);
      endif
```

```
    end
  end
end


% Cleaning temporary variables.
clear mask;
clear i;
clear j;
clear k;
clear countG;
clear count;
clear a;
%===================end of track_file
```

Script "results"

```
%==================start of results
% Written by Miloslav Polasek

% PURPOSE: Calculate average values from the data stored in "track"
matrix.
% A threshold may be applied to filter the data according to the signal
% enhancement observed at 5 minutes after probe injection. This allows
to
% select only well-perfused voxels for the analysis. In our case, we
found
% it useful to define the threshold value as the global (i.e. data for
all
% tested subjects) median of signal enhancement in regions of interest
% at 5 minutes after probe injection.
% Furthermore, counting the number of voxels that surpass the signal
% enhancement threshold or that display positive values of AUC2 allows
% to calculate the fraction of the tissue volume that displays these
% properties, e.g. the fraction of well-perfused tissue volume.

% RESULT: Set of variables containing the calculated average values or
% volume fractions.

% Define the threshold value that characterizes well-perfused voxel.
threshold = 1.0275; % IMPORTANT! Enter a pre-defined number here.

count = 0;
count2 = 0;

% Initialize variables to be calculated.
% Prefix "full" means that all available values are averaged.
% Prefix "thr" means that threshold has been applied and values from
% poorly perfused voxels are excluded from the calculation of average.
thrENH = 0;
thrAUC = 0;
thrAUC2 = 0;
fullENH = 0;
fullAUC = 0;
fullAUC2 = 0;
fullENH2 = 0;
thrENH2 = 0;


% This loop sums the values of individual voxels and counts the
% number of instances. The information will be used to calculate
% average values.
for i=1:size(track,1)
  % Sum all the data (no threshold filtering).
  fullENH = fullENH + track(i,4);
  fullAUC = fullAUC + track(i,5);
  fullAUC2 = fullAUC2 + track(i,6);
  fullENH2 = fullENH2 + track(i,7);

  % Sum only the data in well-perfused voxels.
  if (track(i,4) >= threshold)
    count++; % Count the number of instances.
    thrENH = thrENH + track(i,4);
```

```
    thrAUC = thrAUC + track(i,5);
    thrAUC2 = thrAUC2 + track(i,6);
    thrENH2 = thrENH2 + track(i,7);
  endif

  % Count the number of voxels showing positive AUC2 values.
  if (track(i,6) > 0)
    count2++;
  endif
end

% Calculate the average values.
% Averages of all data (no threshold filtering)
fullENH = fullENH / size(track,1);
fullAUC = fullAUC / size(track,1);
fullAUC2 = fullAUC2 / size(track,1);
fullENH2 = fullENH2 / size(track,1);

% Averages of data filtered according to threshold
% (i.e. only well-perfused voxels).
thrENH = thrENH / count;
thrAUC = thrAUC / count;
thrAUC2 = thrAUC2 / count;
thrENH2 = thrENH2 / count;

% Calculate fractions of the tissue volume with specific properties.
% Volume fraction of well-perfused tissue:
fraction = count / size(track,1);
% Volume fraction of tissue showing positive AUC2 values:
fraction2 = count2 / size(track,1);


% Cleaning temporary variables.
clear i;

%===================end of results
```
